# Serotonergic Modulation Differentially Targets Distinct Network Elements within the Antennal Lobe of *Drosophila melanogaster*

**DOI:** 10.1038/srep37119

**Published:** 2016-11-15

**Authors:** Tyler R. Sizemore, Andrew M. Dacks

**Affiliations:** 1 Department of Biology, West Virginia University, Morgantown, WV, 26505, United States of America

## Abstract

Neuromodulation confers flexibility to anatomically-restricted neural networks so that animals are able to properly respond to complex internal and external demands. However, determining the mechanisms underlying neuromodulation is challenging without knowledge of the functional class and spatial organization of neurons that express individual neuromodulatory receptors. Here, we describe the number and functional identities of neurons in the antennal lobe of *Drosophila melanogaster* that express each of the receptors for one such neuromodulator, serotonin (5-HT). Although 5-HT enhances odor-evoked responses of antennal lobe projection neurons (PNs) and local interneurons (LNs), the receptor basis for this enhancement is unknown. We used endogenous reporters of transcription and translation for each of the five 5-HT receptors (5-HTRs) to identify neurons, based on cell class and transmitter content, that express each receptor. We find that specific receptor types are expressed by distinct combinations of functional neuronal classes. For instance, the excitatory PNs express the excitatory 5-HTRs, while distinct classes of LNs each express different 5-HTRs. This study therefore provides a detailed atlas of 5-HT receptor expression within a well-characterized neural network, and enables future dissection of the role of serotonergic modulation of olfactory processing.

Animals continually alter their behavior to meet dynamic internal and external demands. Neuromodulation promotes behavioral flexibility from anatomically restricted neural networks by altering the biophysical and synaptic properties of individual neurons^1^,^2^. Typically, neuromodulators activate G-protein coupled receptors (GPCRs)^3^ with ligand binding initiating an intracellular signaling cascade that dictates the effect of a neuromodulator on a neuron. Depending on the G-protein associated with a given neuromodulatory receptor, a single neuromodulator can differentially affect the excitability and the synaptic strength of individual neurons in a network^2^,^4^,^5^. Moreover, these receptors can be expressed by multiple cell types within a sensory circuit^6^,^7^,^8^,^9^, and/or concertedly expressed by the same cell^10^, thus compounding the effects of a single neuromodulator. The multi-dimensional effects of a single neuromodulator acting on individual neurons within a network increases the dynamic range of network activity, ultimately promoting depth to behavioral output. Within the antennal lobe (AL) of *Drosophila*, the first olfactory processing center of the brain, the neuromodulator serotonin (5-HT) has widespread effects on odor-evoked responses of different neuronal classes^11^. However, it is difficult to determine how 5-HT modulates olfactory processing without knowing which functional neuron classes express each 5-HT receptor (5-HTR). Here, we exploit recent technological advances to generate a comprehensive atlas of 5-HTR expression in the well-characterized AL of *Drosophila*.

In the AL of *Drosophila* there are three major neuron classes that each perform distinct functions (Fig. 1a); olfactory receptor neurons (ORNs), projection neurons (PNs), and local interneurons (LNs)^12^. The dendrites and soma of odor-detecting ORNs are housed in the antennae and maxillary palps and, generally, each ORN expresses one chemosensory receptor protein endowing them with sensitivity to a particular set of odorants^13^. The axon terminals of ORNs that express the same chemosensory protein converge in the same glomerulus^14^,^15^,^16^ where they form excitatory synapses with PNs and LNs. PN cell bodies surround the AL in 3 distinct cell clusters: ventral, lateral, and anterodorsal cell clusters^17^,^18^. PNs, the second-order neurons of the AL, express acetylcholine (e-PNs)^19^ or GABA (i-PNs)^20^. Recent evidence suggests that these PN types may respond to different categories of odors based on the odor’s attractiveness^21^,^22^,^23^. PNs project to two higher-order brain structures, the mushroom bodies and lateral horn^19^,^24^,^25^. PN spiking activity is refined by several distinct classes of AL LNs that act upon PNs directly, as well as the input that they receive from ORNs and other LNs. LNs are remarkably diverse in their morphology and physiology^26^,^27^. In terms of transmitter content, subsets of LNs express GABA^28^, acetylcholine^29^,^30^, glutamate^31^, neuropeptides^32^,^33^ and can be electrically coupled^34^. Thus, even within these major AL neuron classes, there is a large degree of diversity which may also be indicative of differences in their expression of modulatory receptors.

Within the AL of *Drosophila* there are two 5-HT immunoreactive neurons; the contralaterally projecting, serotonin-immunoreactive deutocerebral (CSD) neurons^35^,^36^. Each CSD neuron innervates both ALs, as well as both lateral horns. Exogenous application of 5-HT in *Drosophila* increases PN sensitivity, and enhances PN responses in an odor-dependent manner^11^. Serotonin also decreases the strength of ORN responses to antennal nerve stimulation by enhancing GABAergic presynaptic inhibition of ORNs. However, 5-HT could enhance the activity of a given neuron by directly affecting excitability or by altering the synaptic input that a neuron receives either by increasing excitation or decreasing inhibition depending on 5-HTR expression within the network. The *Drosophila* genome encodes five 5-HTR genes (5-HT1A, 1B, 2A, 2B, and 7) that target distinct second-messenger pathways. 5-HT1 type, 2 type, and 7 type receptors are negatively coupled to adenylate cyclase, positively coupled to phospholipase C, and positively coupled to adenylate cyclase, respectively^37^,^38^,^39^,^40^. Therefore, the 5-HT1 type receptors are generally inhibitory, while the 5-HT2 type and 7 are generally excitatory^41^. Thus, to determine the receptor basis for the effects of 5-HT on individual neuronal classes within the AL we made use of the newly available 5-HTR MiMIC T2A-GAL4 protein-trap and gene-trap transgenic fly lines^42^ in combination with immunocytochemistry. These fly lines have undergone recombinase-mediated cassette exchange (RMCE) in order to replace their 5′ non-coding (“gene-trap”) or coding-intronic (“protein-trap”) MiMIC cassette with a GAL4 containing cassette^42^,^43^,^44^. In the case of the gene-trap lines, the MiMIC cassette is replaced with a cassette encoding a universal splice-acceptor and GAL4. In the case of the protein-trap lines, the MiMIC cassette is replaced with one that encodes a universal splice acceptor followed by a self-cleaving T2A peptide^45^,^46^ fused to the GAL4 coding sequence (see^42^ for a detailed description of cassette insertion sites). Thus, the gene-trap and protein-trap 5-HTR lines represent endogenous 5-HTR gene transcription and translation, respectively. However, with the exception of 5-HT7, we rely on protein-trap 5-HTR lines to determine what neuronal populations express a given 5-HTR. It should be noted that this approach relies on endogenous 5-HTR translation or transcription to produce GAL4, and subsequently GFP throughout a 5-HTR expressing neuron. Thus, GFP expression does not reflect the distribution of individual 5-HTR proteins along a cell, but rather that a given neuron expressed a 5-HTR. We find that different protein-trap lines for the same 5-HTR highlight neurons of the same functional class (Fig. 1b–f and Table 1). However, in some instances, we note subtle differences between the number of neurons labeled by T2A-GAL4 lines for the same 5-HTR (see Supplementary Information Fig. S1). At the extreme, the difference between two T2A-GAL4 lines for 5-HT2B is ~2–4 PNs out of a population of ~51 PNs (~4–8% of the entire population). More importantly, coding-intronic insertion lines for the same 5-HTR were expressed by the same combinations of neuronal populations (i.e. ORNs, latPNs, vPNs, etc.) and sub-population (i.e. TKKinergic LNs, MIPergic LNs, etc.) (Supplementary Information Fig. S1).

In general, we found that each 5-HTR is expressed by distinct neuronal populations suggesting that 5-HT differentially modulates separate features of olfactory coding. For the most part, the excitatory 5-HTRs (5-HT2A, 2B, and 7) were expressed by excitatory AL neurons, whereas distinct classes of LNs expressed different sets of 5-HTRs. This suggests that 5-HT has both direct effects on PN excitability, as well as indirect effects on PN responses via modulation of the lateral interactions exerted within and between glomeruli by LNs. Our results represent the first steps towards understanding the mechanistic basis for serotonergic modulation on *Drosophila* olfactory processing.

## Results

### Antennae and maxillary palp ORNs express 5-HT2B

**Figure 2:**
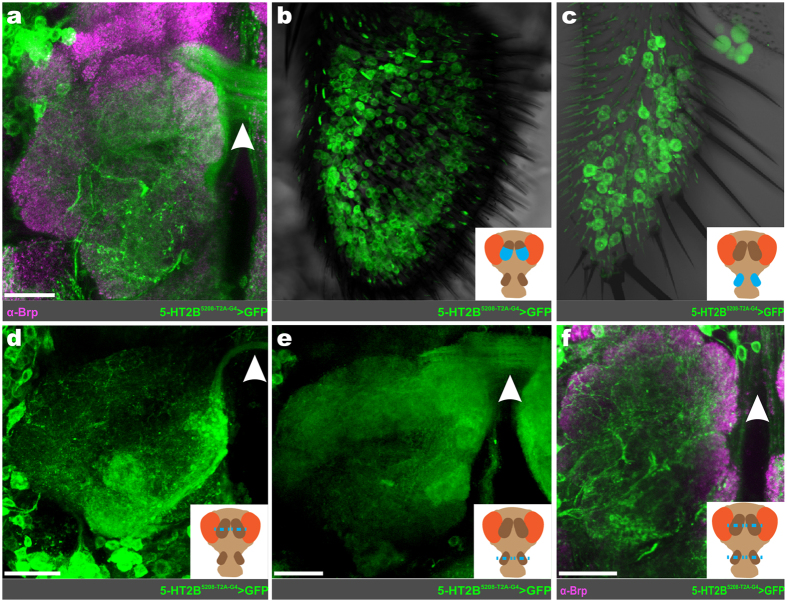
5-HT2B is expressed by antennae and maxillary palp ORNs. (**a**) Representative confocal stack of 5-HT2B expressing ORN axons (green) crossing the AL commissure. (**b**) 5-HT2B expressing ORN soma within the antennae. (**c**) 5-HT2B expressing ORN soma within the maxillary palp. To confirm antennae and maxillary palp ORNs express 5-HT2B, one-day old adults’ antennae (**d**), maxillary palp (**e**), or both (**f**) were removed. Removal of either structure individually only partially abolishes 5-HT2B ORN axons, while removal of both abolishes 5-HT2B ORN axons. The white arrowhead in (**a**) and (**d–f**) highlights ORN axons crossing the AL commissure. Neuropil in (**a**) and (**f**) are delineated by α-Bruchpilot (α-Brp; magenta) labeling. All scale bars = 20 um.

In *Drosophila*, ORN axons cross the midline via the antennal commissure to innervate a specific glomerulus in both the ipsilateral and contralateral AL^47^. Thus, neurites crossing the midline via the antennal commissure provide a reliable anatomical marker for ORNs. We observed a large amount of GFP-expressing fibers crossing through the antennal commissure in both 5-HT2B T2A-GAL4 lines (Fig. 2a) that were not apparent in other 5-HTR lines. The exception to this was the 5-HT7 T2A-GAL4, in which there were a small number of fibers with extremely faint GFP expression (data not shown). Additionally, there were a large number of GFP-expressing cell bodies in both the antennae (Fig. 2b) and the maxillary palps (Fig. 2c), suggesting that ORNs express the 5-HT2B receptor. To confirm that the 5-HT2B T2A-GAL4 driven GFP-expression in axons crossing the antennal commissure originated from ORNs within the antennae and maxillary palps, we ablated either or both appendages of newly eclosed adult flies and examined for the presence of GFP in the antennal commissure. Removal of either the antennae (Fig. 2d) or maxillary palps (Fig. 2e) on their own only partially eliminated the expression of GFP within the antennal commissure. However, removal of both the antennae and maxillary palps resulted in total loss of GFP-positive arbors crossing the antennal commissure (Fig. 2f), indicating that ORNs in both the antennae and maxillary palps express the 5-HT2B receptor.

### Lateral PNs and anterodorsal PNs express excitatory 5-HTRs

**Figure 3:**
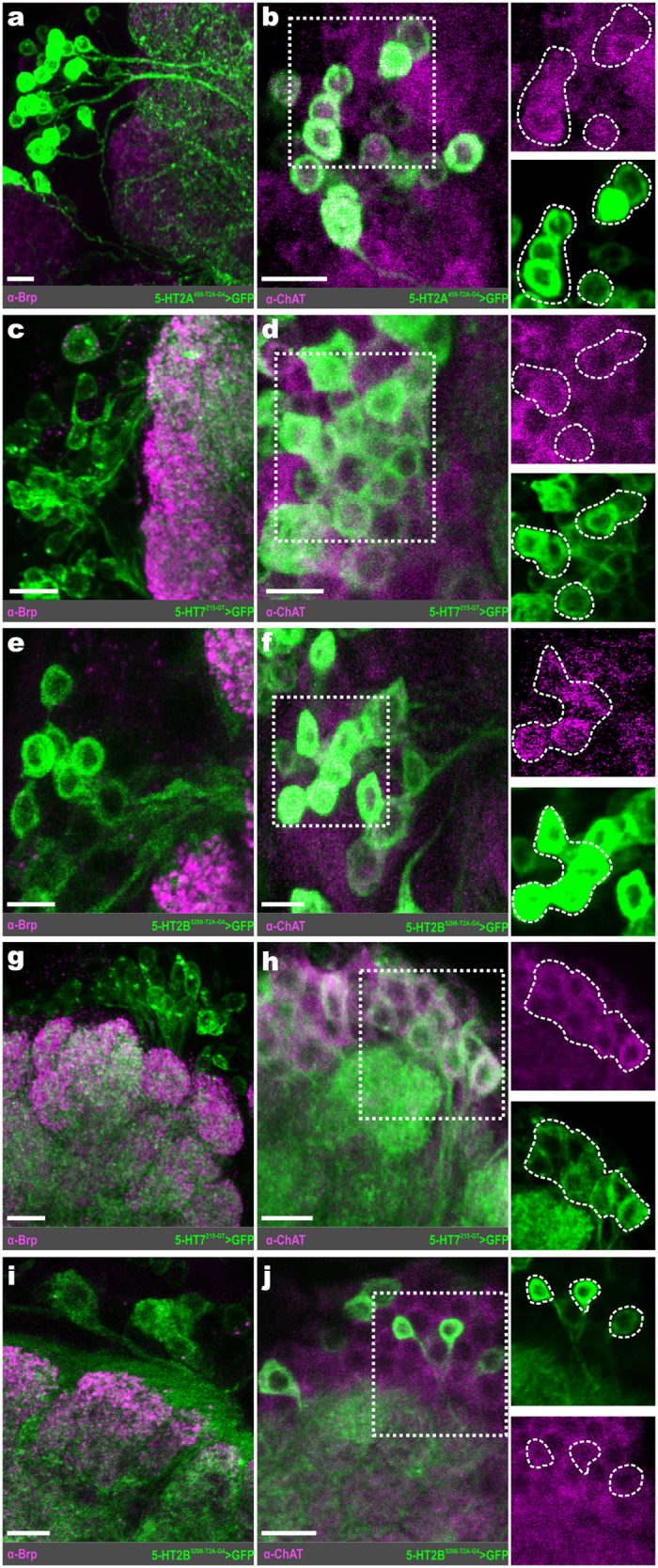
Excitatory PNs express excitatory serotonin receptors. (**a**) Representative confocal stack of 5-HT2A expressing lateral projection neurons (latPNs; green). (**b**) 5-HT2A expressing latPNs also colabel for choline acetyltransferase (ChAT; magenta). (**c**) Representative confocal stack of 5-HT7 expressing latPNs (green). (**d**) 5-HT7 expressing latPNs also co-label for ChAT (magenta). (**e**) Representative confocal stack of 5-HT2B expressing latPNs (green). (**f**) LatPNs that express 5-HT2B also co-label for ChAT (magenta). (**g**) 5-HT7 expressing anterodorsal projection neurons (adPNs; green). (**h**) 5-HT7 expressing adPNs co-label for ChAT (magenta). (**i**) 5-HT2B expressing adPNs (green). (**j**) AdPNs that express 5-HT2B also co-label for ChAT (magenta). Neuropil in (**a**), (**c**), (**e**), (**g**), and (**i**) are delineated by α-Bruchpilot (α-Brp; magenta) labeling. All scale bars = 10 um.

The majority of the excitatory PNs (ePNs) reside in the lateral and anterodorsal cell clusters (latPNs and adPNs, respectively). Previous reports have identified ~35 latPNs^48^ (based on latPNs expressed by GH146-GAL4) and ~73 adPNs^49^, the majority of which are cholinergic^19^. 14.59 ± 1.01 (n = 11) latPNs express 5-HT2A (Fig. 3a,b), while 12.04 ± 0.86 (n = 13) latPNs express 5-HT7 (Fig. 3c,d). The 5-HT2B is expressed by 9.67 ± 0.13 (n* = 2 transgenic lines, n = 13 and 10 brains per line) latPNs (Fig. 3e,f). To a lesser extent, 4.58 ± 0.90 (n* = 3 transgenic lines, n = 6–11 brains per line) latPNs express 5-HT1A (data not shown). Within the adPNs, 19.3 ± 0.53 (n = 10) cells express 5-HT7 (Fig. 3g–h), while 7.88 ± 0.67 (n* = 2 transgenic lines, n = 9 and 7 brains per line) cells express 5-HT2B (Fig. 3i,j). Similar to the number of 5-HT2B expressing adPNs, 5-HT1A is expressed by 8.12 ± 0.62 (n* = 3 transgenic lines, n = 8–9 brains per line) adPNs (data not shown). Exogenous application of 5-HT increases the odor-evoked responses of ePNs within these two cell clusters^11^, therefore this enhancement is at least in part direct in nature, as 5-HT2A and 5-HT7 are positively coupled to IP_3_ and cAMP pathways, respectively.

### Widespread 5-HTR expression within the ventral PNs

Cells of the vPN cell cluster project into the AL through a characteristic fascicle, which we refer to as the “ventral AL fascicle” (see Supplementary Information Fig. S1), and send their axons to the lateral horn through the mediolateral antennal lobe tract (mlALT)^18^. However, the glutamatergic LNs that are ventral to the AL^50^ also project into the AL through the ventral AL fascicle (see Supplementary Information Fig. S2). Therefore, we defined every non-glutamatergic neuron with a soma ventral to the AL that projects into the AL through the ventral AL fascicle as a vPN. Previous reports have identified ~51 vPNs; ~45 vPNs labeled by MZ699-GAL4^49^ and 6 labeled by GH146-GAL4^51^. In terms of transmitter content, ~36 vPNs are GABAergic based on vPNs expressed by GH146-GAL4^24^ and MZ699-GAL4^22^, and while cholinergic vPNs have been described^19^, the number of cholinergic vPNs has not been quantified.

**Figure 4:**
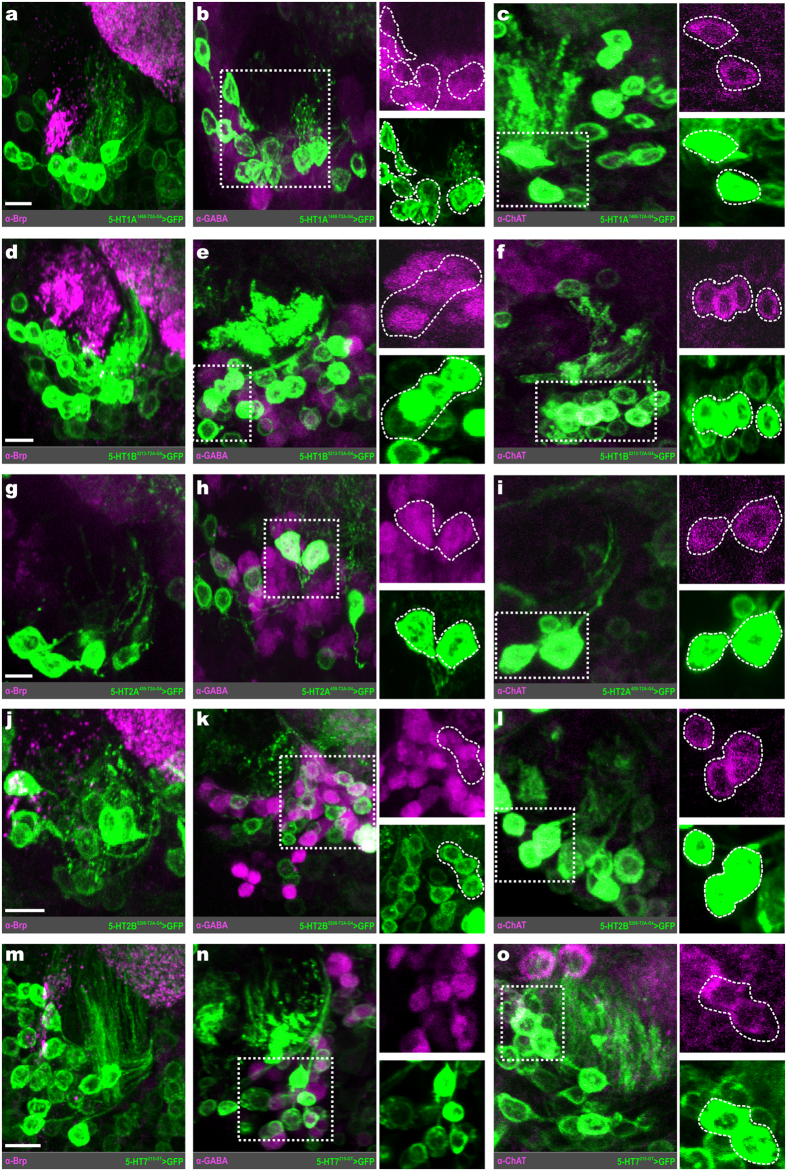
Ventral PNs express each serotonin receptor. (**a**) 5-HT1A is expressed by vPNs (green). (**b**) 5-HT1A expressing vPNs (green) colabel for GABA (magenta). (**c**) 5-HT1A expressing vPNs colabel for choline acetyltransferase (ChAT; magenta). (**d**) 5-HT1B is expressed by vPNs (green). (**e**) 5-HT1B expressing vPNs (green) colabel for GABA (magenta). (**f**) 5-HT1B expressing vPNs colabel for ChAT (magenta). (**g**) 5-HT2A is expressed by vPNs (green). (**h**) 5-HT2A vPNs (green) colabel for GABA (magenta). (**i**) 5-HT2A vPNs (green) colabel for ChAT (magenta). (**j**) 5-HT2B expressing vPNs (green). (**k**) 5-HT2B vPNs (green) colabel for GABA (magenta). (**l**) 5-HT2B vPNs (green) colabel ChAT (magenta). (**m**) vPNs that express 5-HT7 (green). (**n**) 5-HT7 vPNs (green) co-label for GABA (magenta). (**o**) 5-HT7 vPNs (green) colabel for ChAT (magenta). Regions of neuropil in (**a**), (**d**), (**g**), (**j**) and (m) are delineated by α-Bruchpilot (α-Brp; magenta) labeling. All scale bars = 10 um.

Within the vPNs, there are subsets of cells that express each of the 5-HTRs, although the total number of vPNs expressing each receptor did vary between receptor types (Fig. 4). Furthermore, each 5-HTR is expressed by a combination of GABAergic and cholinergic vPNs. The two 5-HT1 type receptors are similarly expressed within the vPNs. 5-HT1A is expressed by 21.70 ± 1.10 (n* = 3 transgenic lines, n = 18–22 brains per line) vPNs, of which 13.84 ± 1.14 are GABAergic and 7.87 ± 0.36 are cholinergic (Fig. 4a–c), while 5-HT1B is expressed by 25.33 ± 1.02 (n = 18) vPNs, of which 15.5 ± 1.02 (n = 9) are GABAergic and 9.83 ± 1.13 (n = 9) are cholinergic (Fig. 4d–f). The 5-HT2A is the least widely expressed receptor within the vPNs, with only 6.73 ± 0.75 (n = 22) vPNs, of which 2.95 ± 0.40 (n = 11) are GABAergic and 3.77 ± 0.69 (n = 11) are cholinergic (Fig. 4g–i). Finally, 5-HT2B and 5-HT7 are expressed in similar numbers of vPNs to the 5-HT1 type receptors. The 5-HT2B is expressed by 19.81 ± 1.30 (n* = 2 transgenic lines, n = 20 and 25 brains per line) vPNs, of which 12.33 ± 1.03 are GABAergic and 7.47 ± 0.75 are cholinergic (Fig. 4j–l). Similarly, 5-HT7 is expressed by 23.55 ± 1.13 (n = 19) vPNs, of which 16.67 ± 2.0 (n = 6) are GABAergic and 6.88 ± 0.52 (n = 13) are cholinergic (Fig. 4m–o). These results suggest that while 5-HT likely has a widespread effect on vPNs, these effects will be heterogeneous as both excitatory and inhibitory 5-HTRs are expressed by both GABAergic and cholinergic vPNs. Moreover, when combined with the observed diversity in 5-HTR expression, these results suggest that the vPN neuronal class is likely more diverse than previously described.

### Distinct populations of LNs express 5-HTRs

The majority of GABAergic cell bodies within the lateral neuroblast cluster are LNs. However, there have been reports of a small number (~1–2) of GABAergic latPNs^20^. Our approach could not objectively distinguish a GABAergic LN from a GABAergic latPN. Therefore, we make the assumption that GABAergic cell bodies in the lateral cell cluster are likely LNs, knowing that there are a small number of GABAergic latPNs^20^. However, there is a small subset of cholinergic LNs ventrolateral to the AL that are easily discernable, based on soma size, from cholinergic PNs^34^. There are ~200 LNs within the AL^52^ that express a diverse array of transmitters^27^ including glutamate^50^ and neuropeptides such as tachykinin (TKK) and myoinhibitory peptide (MIP)^32^,^33^, and vary in their synaptic connectivity with other AL neuron classes^30^,^34^,^53^,^54^.

**Figure 5:**
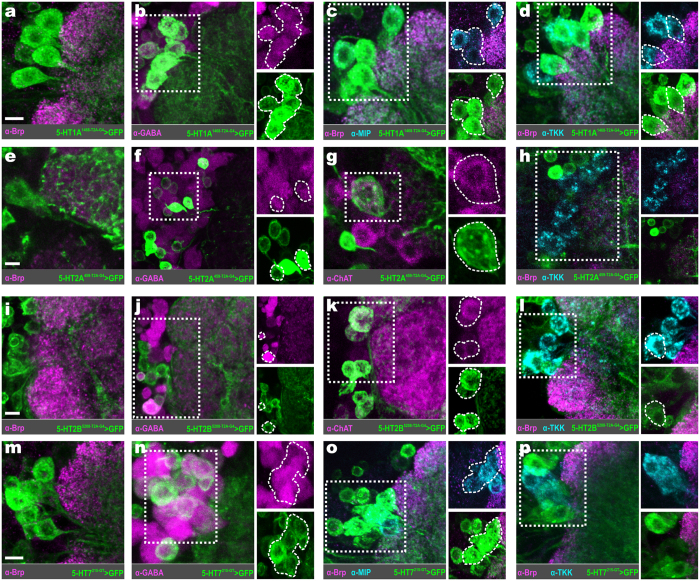
5-HTRs are expressed by distinct populations of local interneurons. (**a**) Local interneurons (LNs) that express 5-HT1A. (**b**) 5-HT1A LNs (green) colabel for GABA (magenta). (**c**) 5-HT1A LNs (green) colabel for myoinhibitory peptide (MIP; cyan). (**d**) 5-HT1A LNs colabel for tachykinin (TKK; cyan). (**e**) 5-HT2A expressing LNs (green). (**f**) 5-HT2A LNs (green) colabel for GABA (magenta). (**g**) Cholinergic LNs (ChAT; magenta) express 5-HT2A. (**h**) 5-HT2A (green) is not expressed by any TKKinergic LN (cyan). (**i**) LNs that express 5-HT2B. (**j**) 5-HT2B LNs (green) colabel for GABA (magenta). (**k**) Cholinergic LNs (ChAT; magenta) express 5-HT2B. (**l**) 5-HT2B (green) is expressed by TKKinergic LNs (cyan). (**m**) 5-HT7 expressing LNs. (**n**) 5-HT7 LNs colabel for GABA (magenta). (**o**) 5-HT7 LNs colabel for MIP (cyan). (**p**) 5-HT7 LNs colabel for TKK (cyan). Neuropil in (**a**), (**c–e**), (**i**), (**l**), (**m**), and (**o**,**p**) are delineated by α-Bruchpilot (α-Brp; magenta) labeling. All scale bars = 10 um.

Distinct subcategories of LNs, based on transmitter content, express distinct 5-HTRs (Fig. 5). The 5-HT1A is expressed by 13.63 ± 0.55 (n* = 3 transgenic lines, n = 17–18 brains per line) lateral LNs (Fig. 5a) whose cell bodies are consistently located in close proximity to the AL. Of these LNs, 12.40 ± 0.86 are GABAergic (Fig. 5b). In addition, a significant proportion of the 5-HT1A expressing LNs are peptidergic, with 8.62 ± 0.01 LNs expressing MIP (Fig. 5c) and 7.00 ± 0.26 expressing TKK (Fig. 5d). The 5-HT2A receptor is expressed by a smaller number of lateral LNs (4.27 ± 0.96, n = 11) relative to the 5-HT1A LNs (Fig. 5e). Of these, 3.73 ± 0.81 (n = 11) are GABAergic (Fig. 5f), 1.14 ± 0.10 (n = 11) are cholinergic (Fig. 5g), and none of the 5-HT2A LNs were TKKinergic (Fig. 5h). The 5-HT2B expressing lateral LNs (Fig. 5i), of which there are 12.42 ± 0.93 (n* = 2 transgenic lines, n = 25 and 20 brains per line), are primarily GABAergic (10.12 ± 0.78; Fig. 5j), although 2.30 ± 0.15 are cholinergic (Fig. 5k), and roughly a single (0.87 ± 0.68) TKKinergic LN (Fig. 5l). Finally, the 5-HT7 receptor is also expressed by lateral LNs (Fig. 5m; 12.19 ± 0.71, n = 16) which are predominantly GABAergic (Fig. 5n; 11.25 ± 0.69, n = 8), although a small number (4.64 ± 0.21, n = 7) are MIPergic (Fig. 5o) and 1.72 ± 0.15 (n = 9) TKKinergic (Fig. 5p).

**Figure 6:**
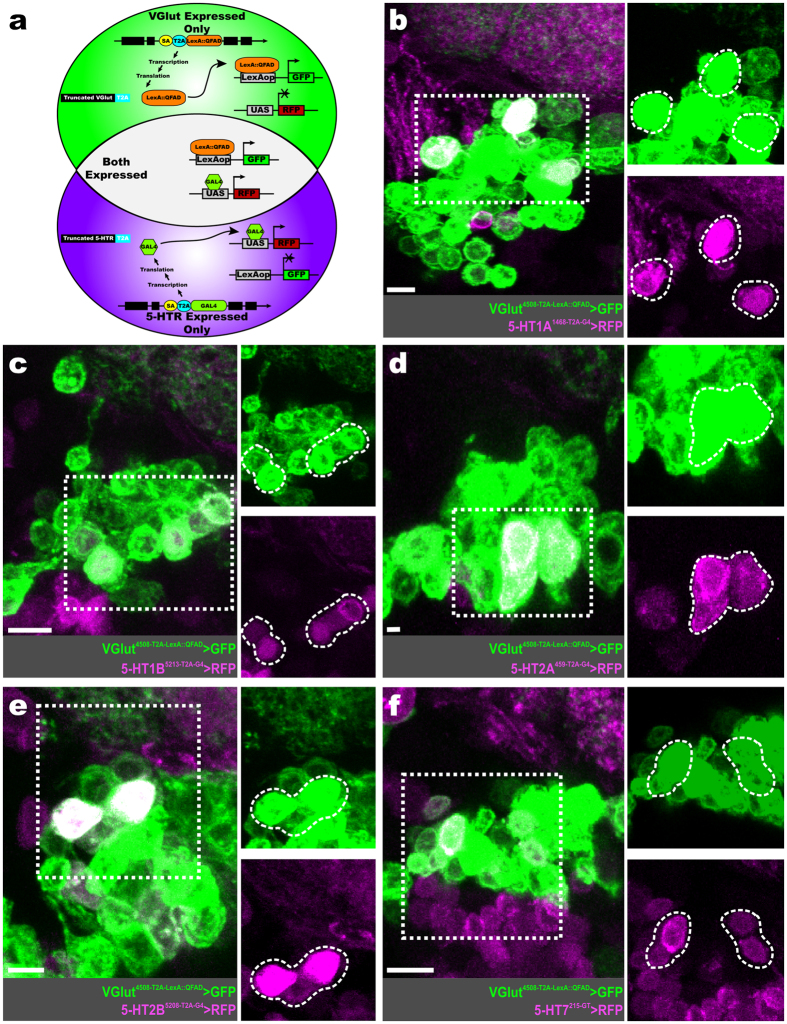
Glutamatergic LNs express each 5-HTR. (**a**) Schematic of approach used to determine 5-HTR expression within glutamatergic LNs. We used a Trojan T2A-LexA::QFAD protein-trap line for vesicular glutamate transporter (VGlut) to drive the expression of GFP (green) in every glutamatergic neuron. Within the same animal, RFP (magenta) is produced in every neuron that produces a given 5-HTR, depending on the 5-HTR T2A-GAL4 line used. Both GFP and RFP (white) is produced in glutamatergic neurons that express a given 5-HTR. (**b**) Glutamatergic LNs (green) that co-express the 5-HT1A (magenta). (**c**) Glutamatergic LNs (green) that co-express the 5-HT1B (magenta). (**d**) Glutamatergic LNs (green) that co-express the 5-HT2A (magenta). (**e**) Glutamatergic LNs (green) that co-express the 5-HT2B (magenta). (**f**) Glutamatergic LNs (green) that co-express the 5-HT7 (magenta). All scale bars = 10 um.

To assess 5-HTR expression within the glutamatergic LNs that are ventral to the AL^50^, we performed RFP-GFP dual-expression experiments. In these cases, a Trojan-LexA::QFAD protein-trap line for vesicular glutamate transporter (VGlut) was used to produce GFP in all cells that produce VGlut^55^, simultaneously RFP is produced in all cells that produce a given 5-HTR via the T2A-GAL4 5-HTR driver (Fig. 6a). The 5-HT1A is expressed by 7.75 ± 0.43 (n* = 5-HT1A^1468-T2A-G4^, n = 10 brains) glutamatergic LNs (Fig. 6b). Similarly, the 5-HT1B is expressed by 6.89 ± 0.48 (n = 9 brains) glutamatergic LNs (Fig. 6c). The 5-HT2A is expressed by 3.72 ± 0.19 (n = 9 brains) glutamatergic LNs (Fig. 6d), while 5-HT2B is expressed by 9.75 ± 0.40 (n* = 5-HT2B^5208-T2A-G4^, n = 10 brains) glutamatergic LNs (Fig. 6e). Finally, 5-HT7 is expressed by 9.50 ± 0.54 (n = 8 brains) glutamatergic LNs (Fig. 6f).

## Discussion

**Figure 7:**
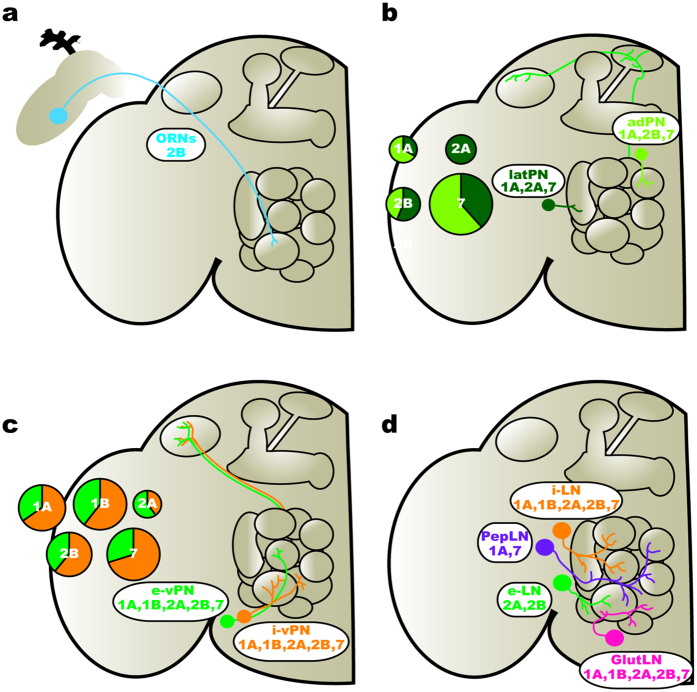
Serotonin targets distinct network elements within the AL. (**a**) ORNs (blue) within the antennae and maxillary palps express 5-HT2B. (**b**) Excitatory PNs (ePNs) in the lateral (latPNs; dark green) and anterodorsal (adPNs; lime green) clusters express 5-HT2A and 5-HT7, respectively. In all cases, the pie chart diameter represents the total number of ePNs that express a given 5-HTR, and is divided by the relative number of latPNs (dark green slices) and adPNs (lime green slices) that express a given 5-HTR. (**c**) Inhibitory ventral PNs (i-vPN; orange) and excitatory ventral PNs (e-vPN; green) express all 5-HTRs. In all cases, the pie chart diameter represents the total number of vPNs that express a given 5-HTR. Moreover, each pie chart is divided by the relative number of i-vPNs (orange slices) and e-vPNs (green slices) that express a given 5-HTR. (**d**) GABAergic local interneurons (i-LN; orange) express all 5-HTRs. Cholinergic LNs (e-LN; green) express 5-HT2A and 2B. Peptidergic LNs (PepLN; purple) express 5-HT1A and 5-HT7. Glutamatergic LNs (GlutLN; pink) express all 5-HTRs.

Neuromodulators often act through diverse sets of receptors expressed by distinct network elements and in this manner, differentially affect specific features of network dynamics. Knowing which network elements express each receptor for a given neuromodulator provides a framework for making predictions about the mechanistic basis by which a neuromodulator alters network activity. In this study, we provide an “atlas” of 5-HTR expression within the AL of *Drosophila*, thus revealing network elements subject to the different effects of serotonergic modulation. In summary, we find that different receptors are predominantly expressed by distinct neuronal populations (Fig. 7a–d). For example, the 5-HT2B is expressed by ORNs (Fig. 7a), while the 5-HT2A and 7 are expressed by cholinergic PNs (Fig. 7b). Additionally, we find that each receptor is expressed by diverse populations of LNs, with the exception the 5-HT1B. For instance, 5-HT1A is expressed by GABAergic and peptidergic (TKK and MIP) LNs, while 5-HT2A and 2B are not expressed by peptidergic LNs (Fig. 7d). However, the vPNs are the exception to the general observation that distinct neuronal classes differ from each other in the 5-HTRs (Fig. 7c) and we discuss the implications of this below. Together, our results suggest that within the AL, 5-HT differentially modulates distinct populations of neurons that undertake specific tasks in olfactory processing.

A recurring theme of neuromodulation is that the expression of distinct receptor types by specific neural populations allows a single modulatory neuron to differentially affect individual coding features. For instance, GABAergic medium spiny neurons (MSNs) in the nucleus accumbens express either the D1 or D2 dopamine receptor allowing dopamine to have opposite effects on different MSNs via coupling to different G_alpha_ subunits (reviewed in^56^). MSNs that differ in dopamine receptor expression also differ in their synaptic connectivity. Dopamine activates D1-expressing MSNs that directly inhibit dopaminergic neurons in the ventral tegmental area (VTA), and inhibits D2-expressing MSNs that inhibit GABAergic VTA interneurons thus inducing suppression of dopamine release. In this manner, a single neuromodulator differentially affects two populations of principal neurons via different receptors to generate coordinated network output. This principle also holds true for the effects of 5-HT within the olfactory bulb. For instance, 5-HT enhances presynaptic inhibition of olfactory sensory neurons by 5-HT2C-expressing juxtaglomerular cells^57^, while increasing excitatory drive to mitral/tufted cells and periglomerular cells via 5-HT2A-expressing external tufted cells^58^. Similarly, we observed that distinct classes of AL neurons differ in their expression of 5-HTRs. For instance, ePNs express the 5-HT2A, 5-HT2B and 5-HT7 receptors (Fig. 3), while peptidergic LNs predominantly express the 5-HT1A receptor (Fig. 5c,d). This suggests that the cumulative effect of 5-HT results from a combination of differential modulation across neuronal populations within the AL. Interestingly, although we find that 5-HT2B is expressed by ORNs, previous reports found that 5-HT does not directly affect *Drosophila* ORNs^11^. In this study, ORNs were stimulated using antennal nerve shock in which the antennae were removed in order to place the antennal nerve within a suction electrode^11^. Thus, if 5-HT2B is localized to the ORN cell body, removal of the antennae would eliminate any effect of 5-HT on ORNs. In several insects, 5-HT within the antennal haemolymph modulates ORN odor-evoked responses^59^,^60^. Therefore, it is plausible ORNs are modulated by a source of 5-HT other than the CSD neurons within the AL.

Serotonergic modulation of LN activity has widespread, and sometimes odor specific, effects on olfactory processing. LNs allow ongoing activity across the AL to shape the activity of individual AL neurons, often in a glomerulus specific manner creating non-reciprocal relationships^53^,^61^,^62^,^63^. It is fairly clear that 5-HT directly modulates LNs, although 5-HT almost certainly affects synaptic input to LNs. Serotonin modulates isolated *Manduca sexta* LNs *in vitro*^64^ and, consistent with our results, a small population of GABAergic LNs in the AL of *Manduca* also express the 5-HT1A receptor^65^. Furthermore, 5-HT has odor-dependent effects on PN odor-evoked activity^11^,^66^, suggesting that odor specific sets of lateral interactions are modulated by 5-HT. We found that different populations of LNs expressed different sets of 5-HT receptors, however we categorized LNs based on transmitter type, so it is possible that these categories could be even further sub-divided based on morphological type, synaptic connectivity or biophysical characteristics^26^,^27^,^30^,^34^. Regardless, our results suggest that 5-HT modulates lateral interactions within the AL by selectively affecting LN populations that undertake different tasks. For instance, the TKKergic LNs that express the 5-HT1A receptor provide a form of gain control by presynaptically inhibiting ORNs^32^. Our results suggest that 5-HT may affect TKK mediated gain control differently relative to processes undertaken by other LN populations. Furthermore, the expression of the TKK receptor by ORNs is regulated by hunger, allowing the effects of TKK to vary with behavioral state^67^. It would be interesting to determine if the expression of 5-HTRs themselves also vary with behavioral state as a means of regulating neuromodulation within the olfactory system.

Although we primarily found that individual populations of AL neurons chiefly expressed a single or perhaps two 5-HTR types, the vPNs appear to be an exception. As a population, the vPNs express all of the 5-HTRs (Fig. 4) and the vPNs that express each 5-HTR did not appear to differ in terms of the proportion of those neurons that were GABAergic or cholinergic (roughly 3:2). Unfortunately, our approach does not allow us to determine the degree to which individual vPNs co-express 5-HTRs. However, it is estimated that there are ~51 vPNs and even if this is an underestimate, there is likely some overlap of receptor types as a large number of vPNs expressed the 5-HT1A, 1B, 2B and 7 receptors. It is possible that a single vPN expresses one 5-HTR in the AL and a different 5-HTR in the lateral horn. However, our approach only allows us to identify which neurons express a given 5-HTR, not where that receptor is expressed. The CSD neurons ramify throughout both ALs and both lateral horns^35^,^36^, thus vPNs could have differential spatial expression of individual 5-HTRs. Individual neurons expressing multiple 5-HTRs has been demonstrated in several neural networks. For instance, pyramidal cells in prefrontal cortex express both the 5-HT1A and 5-HT2A^7^. This allows 5-HT to have opposing effects that differ in their time course in the same cell^9^,^10^,^68^. In terms of the vPNs, our results suggest that the current understanding of the diversity of this neuron class is limited. The expression of receptors for different signaling molecules could potentially be a significant component to vPN diversity.

Neuromodulators are often released by a small number of neurons within a network, yet they can have extremely diverse effects depending upon patterns of receptor expression. For the most part, individual populations of AL neurons differed in the receptor types that they expressed. This suggests that 5-HT differentially acts on classes of neurons that undertake distinct tasks in olfactory processing. In the case of the vPNs, this differential modulation may be fairly complex due to the diversity within this neuronal class. Our goal was to establish a functional atlas of 5-HTR expression in the AL of *Drosophila*. This dataset therefore provides a mechanistic framework for the effects of 5-HT on olfactory processing in this network.

## Methods

### Fly Stocks

Flies were maintained on standard cornmeal at 24 °C and under a 12:12 light:dark cycle. MiMIC T2A-GAL4 protein- and gene-trap stocks were graciously provided by Dr. H.A. Dierick and have been previously described^42^. These include: 5-HT1A-T2A-GAL4^MI04464^, 5-HT1A-T2A-GAL4^MI01140^, 5-HT1A-T2A-GAL4^MI01468^, 5-HT1B-T2A-GAL4^MI05213^, 5-HT2A-T2A-GAL4^MI00459^, 5-HT2B-T2A-GAL4^MI05208^, 5-HT2B-T2A-GAL4^MI06500^, and 5-HT7-GAL4^MI00215^. All 5-HT receptor protein-trap and gene-trap lines were crossed to membrane-targeted UAS-IVS-mCD8::GFP^69^ (BL32185). Dr. Tzumin Lee kindly provided the MZ699-GAL4 and GH146-LexA stocks^49^. The Trojan-LexA::QFAD VGlut protein-trap line^55^ (BL60314) recombined with y, w,10xUAS-RFP, LexAop-GFP (BL32229) was generously provided by Dr. Quentin Gaudry.

### Immunocytochemistry

Brains were dissected in *Drosophila* external saline (CSHL recipe) fixed in 4% paraformaldehyde for 30 minutes on ice, washed with phosphate buffered saline with 0.5% Triton-X 100 (PBST), and blocked for 1 hour in PBST with either 2% IgG-free BSA (Jackson Immunoresearch; Cat#001-000-162), or 5% NGS (for GABA & ChAT labeling; Jackson Immunoresearch; Cat#005-000-121). In many instances, an ascending-descending ethanol wash series (30%, 50%, 70%, 95%, 100%, 95%, 70%, 50%, 30%) was used prior to blocking to clear air from residual trachea. Brains were incubated at 4 °C in primary antibody diluted with blocking solution and 5mM sodium azide. Primary antibody dilutions used include: 1:50 mouse anti-Bruchpilot (DSHB; mAbnc82^70^), 1:500 rabbit anti-GABA (Sigma; Cat#A2052), 1:200 mouse anti-ChAT (DSHB; ChAT4B1^71^), 1:5,000 rabbit anti-TKK (provided by Dr. Jan Veenstra^72^), 1:4,000 rabbit anti-MIP (provided by Dr. Christian Wegener^73^), and 1:1,000 rabbit anti-GFP (Life Technologies; Cat#A-11122). Brains were then washed in PBST, blocked as above, and incubated at 4 °C in secondary antibody diluted with blocking solution and 5mM sodium azide. All secondary antibodies were purchased from Life Technologies and include: goat anti-rabbit Alexa-488 (Cat#A-11008), donkey anti-rabbit Alexa-488 (Cat#A-21206), donkey anti-mouse Alexa-546 (Cat#A-10036), goat anti-mouse Alexa-546 (Cat#A-11030), goat anti-rabbit Alexa-633 (Cat#A-21070), and goat anti-mouse Alexa-633 (Cat#A-21050). Brains were then washed in PBST and PBS, then cleared via an ascending glycerol series (40%, 60%, 80%), and finally mounted on well slides in Vectashield^®^ (Vector Laboratories, Burlingame, CA; Cat#H-1200).

### Image Acquisition and Analysis

Brains were imaged using an Olympus BX61 (Shinjuku, Tokyo, Japan) confocal microscope running the Fluoview FV1000 software with a 40x UPlanFL-N or 60x PlanApo-N oil-immersion objective. In some cases, brightness and contrast were manually adjusted in Adobe Photoshop v.14.2 (San Jose, CA). GFP-positive and additional primary labeled cell bodies were recorded in VAA3D (v.3.20)^74^. Anterodorsal, lateral, and ventral PN and LNs were defined by cell body location^49^ and, in the case of the lateral PNs and LNs, transmitter content.

### ORN Ablations

To demonstrate that 5-HT2B is expressed in both antennae and maxillary palp ORNs, the antennae, maxillary palps, or both were removed 4–5 hours post-eclosion. Animals were kept under standard conditions and media until 10-days later when they were processed for immunocytochemistry.

### Statistical Analysis

All statistics were performed in GraphPad Prism v.6.01 (GraphPad Software, La Jolla, CA). For simplicity, we use the average of the averages for multiple transgenic lines used for the same receptor (i.e. 5-HT1A and 5-HT2B) when reporting results. In these cases, data are presented as mean of the mean of each individual line ± mean of the s.e.m of each individual line (n* = total number of transgenic lines for a receptor, n = total number of brains for each transgenic line). All other data are presented as mean ± s.e.m (n = total number of brains). A D’Agostino and Pearson omnibus normality test (α = 0.05) was used to confirm normal distribution of neuronal classes highlighted between the multiple lines for 5-HT1A and 2B. A one-way ANOVA followed by a Tukey’s multiple comparison test (α = 0.05) was used to test for significant differences between the number of neurons within a neuronal class highlighted by the different 5-HT1A T2A-GAL4 lines. An unpaired Student’s *t*-test (α = 0.05) was performed to test for significant differences between the number of neurons within a neuron class highlighted by the different 5-HT2B T2A-GAL4 lines for the same 5-HTR.

## Additional Information

**How to cite this article**: Sizemore, T. R. and Dacks, A. M. Serotonergic Modulation Differentially Targets Distinct Network Elements within the Antennal Lobe of *Drosophila melanogaster. Sci. Rep.* **6**, 37119; doi: 10.1038/srep37119 (2016).

**Publisher’s note:** Springer Nature remains neutral with regard to jurisdictional claims in published maps and institutional affiliations.

## Supplementary Material

Supplementary Information

## Figures and Tables

**Figure 1:**
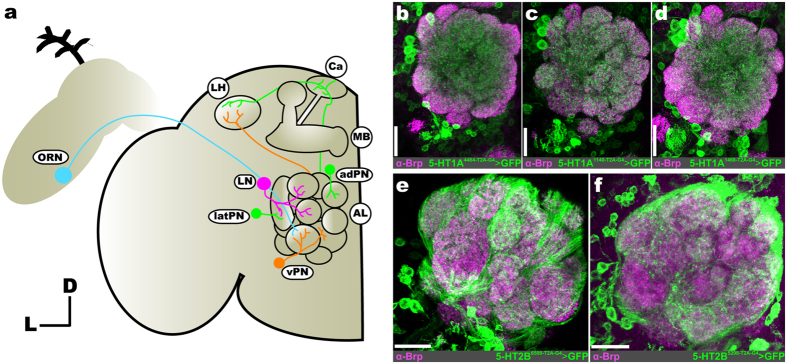
Consistent neuron labeling from different coding-intronic insertions of 5-HTRs in the antennal lobe of Drosophila melanogaster. (**a**) Olfactory receptor neurons (ORNs; cyan) housed within the antennae and maxillary palps (not depicted here) send axons to a single glomerulus in the antennal lobe (AL). Within a glomerulus, ORNs synapse on projection neurons (PNs; green and orange) and local interneurons (LNs; magenta). LNs interconnect glomeruli and synapse on ORNs, PNs, and other LNs. A given PN is classified as an anterodorsal PN (adPN; green), lateral PN (latPN; green), or ventral PN (vPN; orange) based on its cell body position. PN axons project to the mushroom body (MB) calyx (Ca) and lateral horn (LH). Ellipses indicate neuron type, while circles indicate specific brain regions. (**b–d**) T2A-GAL4 conversion of three separate MiMIC insertions (4464, 1140, and 1468, respectively) in the 5-HT1A locus reveals consistent labeling of LNs and vPNs. (**e** and **f**) T2A-GAL4 conversion of two separate MiMIC insertions (6500 and 5208, respectively) in the 5-HT2B locus consistently labels ORNs. Neuropil in (**b–f**) are delineated by α-Bruchpilot (α-Brp; magenta) labeling. All scale bars = 20 um.

**Table 1:**
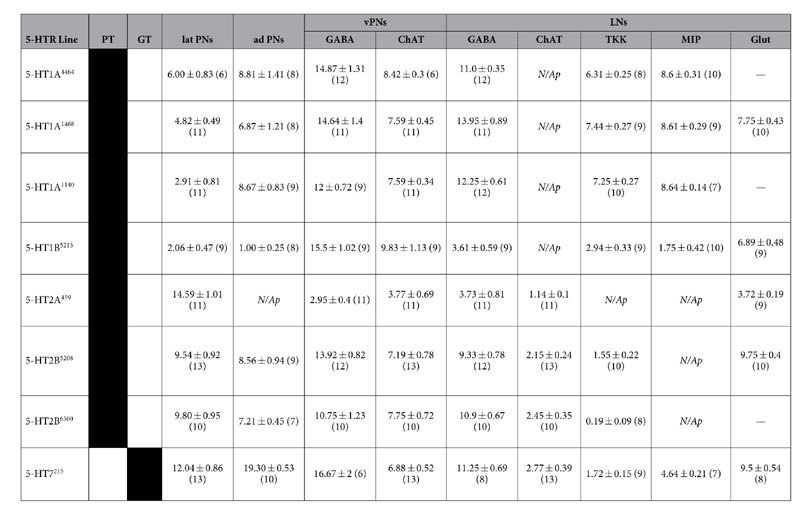
5-HTR MiMIC T2A-GAL4 transgenic lines used and the number of cells in each cluster that express each receptor.

With the exception of 5-HT7, our investigation relies solely on MiMIC T2A-GAL4 protein-trap transgenics (PT). In all cases, the number of cells in each cluster that express each receptor are represented as mean ± s.e.m. (n  =  number of brains). Note that the total number of LNs that express a given 5-HTR line is the total of the GABA, cholinergic (ChAT), and glutamatergic (Glut) LN columns, since peptidergic LNs are also GABAergic. “PT” and “GT” describe which lines are protein-traps and which are gene-trap, respectively. “*N/Ap*” denotes 5-HTR expressing neuron classes that did not co-label for that given transmitter, or expressed by that neuronal class. Dashes (“—”) denote lines that were not tested for colabeling.
