# Supplementary material for: Serotonergic Modulation Differentially Targets Distinct Network Elements within the Antennal Lobe of Drosophila melanogaster
Source: Sci Rep. 2016 Nov 15;6:37119. doi: 10.1038/srep37119 (PMC5109230; doi:10.1038/srep37119)
Supplement: Supplementary Information [file srep37119-s1.pdf]

Supplemental Information for

Serotonergic Modulation Differentially Targets Distinct Network Elements within the Antennal Lobe of Drosophila melanogaster

Tyler R. Sizemore[1] and Andrew M. Dacks[1*]

Institutional affiliations: [1]Department of Biology, West Virginia University, Morgantown, WV, 26505, United States of America

* Corresponding author: Tel: 304-293-3205; Fax: 304-293-6363,
E-mail: andrew.dacks@mail.wvu.edu

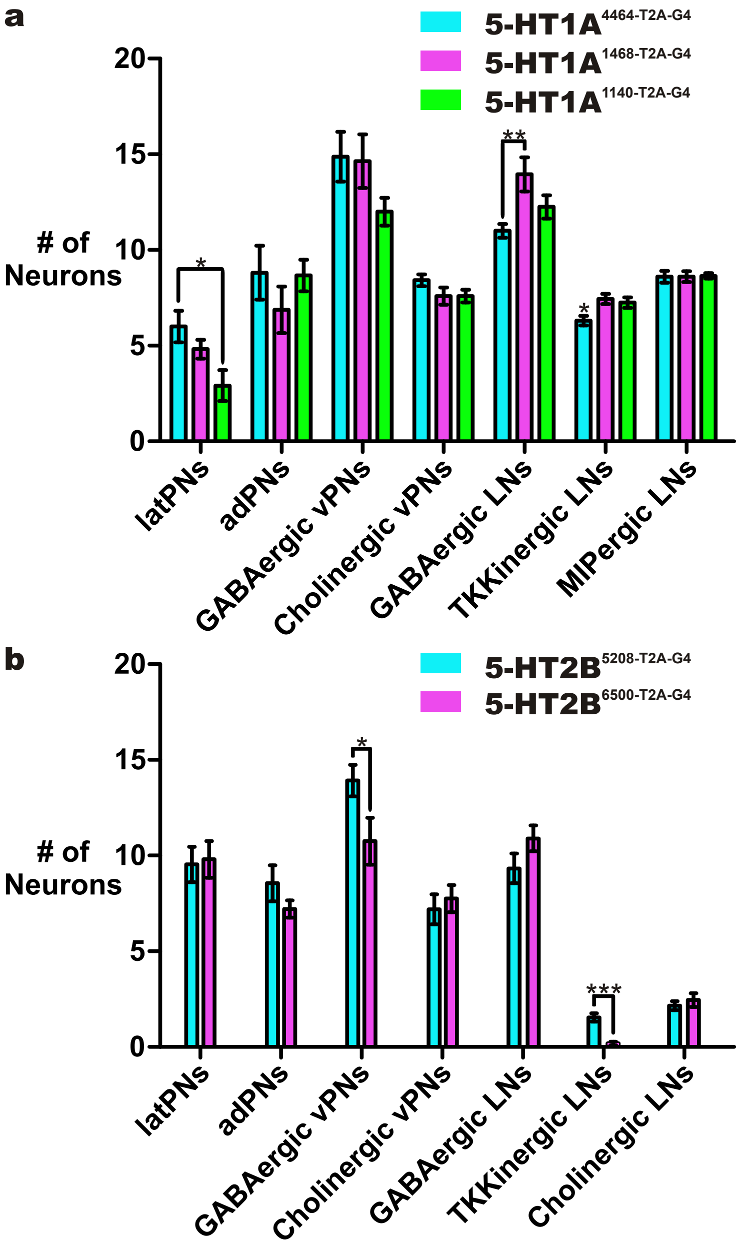

Supplementary Figure 1 Different coding-intronic insertion drivers for the same locus label similar neurons. (a) Number of latPNs, adPNs, GABAergic vPNs, cholinergic vPNs, TKKinergic LNs, and MIPergic LNs highlighted by each T2A-GAL4 line for 5-HT1A. Data were compared using a 1-Way ANOVA followed by a Tukey's Multiple Comparison Test (alpha=0.05). (b) Number of latPNs, adPNs, GABAergic vPNs, cholinergic vPNs, TKKinergic LNs, and cholinergic LNs highlighted by each T2A-GAL4 line for 5-HT2B. The difference between the number of TKKinergic LNs highlighted by these two lines is one neuron. The number of glutamatergic LNs labeled by each transgenic line was not tested. Data were compared using an unpaired Student's *t*-test (alpha=0.05). In all graphs, error bars are s.e.m. $*p < 0.05$; $**p < 0.005$; $***p < 0.0005$.

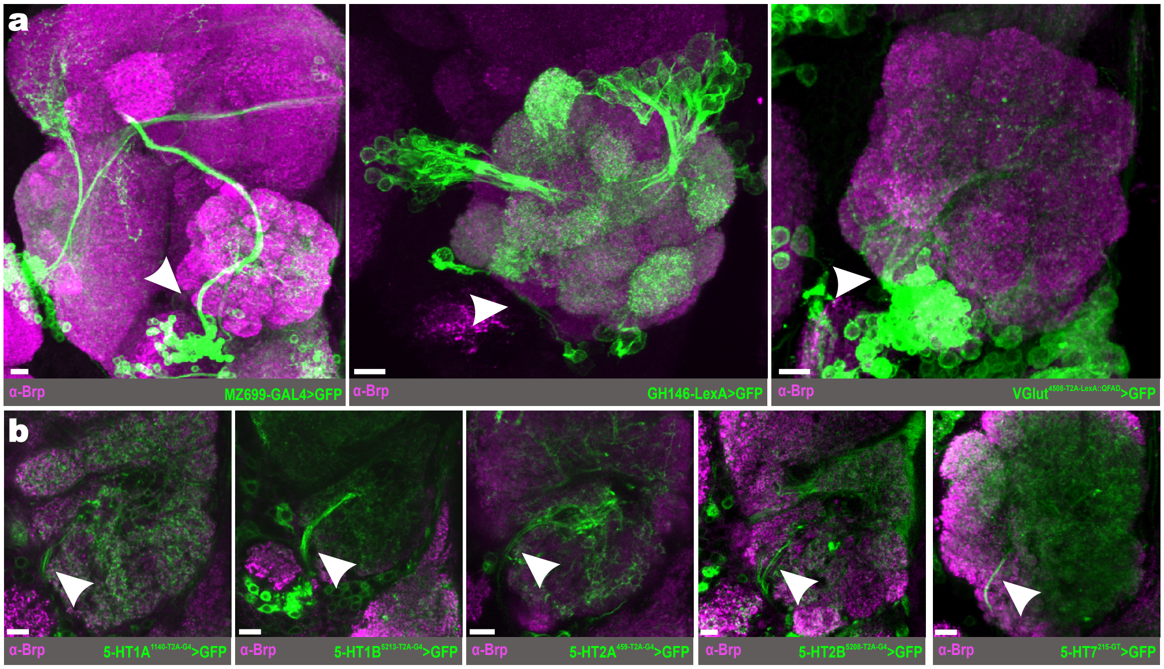

Supplementary Figure 2 vPNs and glutamatergic LNs enter the AL through the ventral AL fascicle. (a) Representative confocal stack of the MZ699 enhancer-trap GAL4, GH146 enhancer-trap LexA, and a T2A-LexA::QFAD protein-trap line for vesicular glutamate (VGlut) driving the expression of GFP (green). MZ699-GAL4 and GH146-LexA ventral PNs, as well as glutamatergic LNs, project into the AL through the ventral AL fascicle. (b) Representative confocal stack of the T2A-GAL4 MiMIC 5-HTR lines driving the expression of GFP (green). Similar to the neurites of MZ699-GAL4, GH146-LexA, and T2A-LexA::QFAD VGlut, 5-HTR expressing ventral neurons enter the AL through the ventral AL fascicle. In all cases, the white arrowhead demarcates the ventral AL fascicle. Neuropil is delineated by α-Bruchpilot (α-Brp; magenta) labeling. All scale bars=10um.
